# Child abuse in the West Bank of the occupied Palestinian territory (WB/oPt): social and political determinants

**DOI:** 10.1186/s12889-020-09251-x

**Published:** 2020-07-18

**Authors:** Nouh Harsha, Margaret A. Lynch, Rita Giacaman

**Affiliations:** 1 Doctoral School of Health Sciences, University of Debrecen, Debrecen, Hungary; 2 Institute of Community and Public Health, Birzeit University- ICPH/BZU, Birzeit, Palestine; 3 Kings College, London, UK; 4 ICPH/BZU, Birzeit, Palestine

**Keywords:** Child abuse, Physical and emotional abuse, Education levels, West Bank, Parental warmth, Political violence

## Abstract

**Background:**

Child maltreatment is a global epidemic. It affects morbidity, mortality, social behavior, wellbeing, and quality of life of children. This study aims to assess prevalence of child abuse in the West Bank (WB) of the occupied Palestinian territory (oPt) and to determine some of its social and political associated factors.

**Methods:**

We analyzed secondary data obtained from a cross sectional study conducted on a sample representing Palestinian children on the West Bank and using the International Society for the Prevention of Child Abuse and Neglect (ISPCAN) tool. The ISPCAN Child Abuse Screening Tool for parents (ICAST-P) questionnaire was completed by 1107 Palestinian mothers to estimate physical and emotional child abusive practices at home for children aged 0–12 years. Univariate, bivariate, and multivariate binary logistic regression analyses were performed using the SPSS® version 20 to assess prevalence and predictors of child abuse.

**Results:**

Overall, around 34% of the West Bank-children were abused by their mothers. Results of the logistic regression analysis indicated that male children, children of younger mothers, children whose fathers were with low levels of education, children whose mothers reported low levels of parental warmth, and children whose parents were exposed to political violence were at greater risk of being abused.

**Conclusions:**

Child abuse is highly prevalent among children of the Palestinian society in the West Bank. Policy makers need to pay more attention to this epidemic. The association between child abuse and political violence found in this study makes a just solution for Palestinians essential for improving the welfare of children and families.

## Background

In 2008, the Centres for Disease Control and Prevention defined child maltreatment as “Any act or series of acts of commission or omission by a parent or other caregiver that results in harm, potential for harm, or threat of harm to a child” [1]. Commissions refer to child abuse including physical, sexual, and psychological (emotional) abuse, and omissions refer to neglect whether physical, emotional, medical, or educational [2].

Child abuse is a global health concern [3–5], occurring in both developing and developed nations [6, 7]. A systematic review on global prevalence of violence against children in 96 countries reported that over one billion of children had experienced physical, emotional or sexual violence during the year that preceded the survey [8]. Likewise, the United Nations International Children’s Emergency Fund (UNICEF) reported that around 300 million children aged 2 to 4 years are subjected to violent disciplines around the world [9]. In the USA, 80% of the children were spanked by the time they were in the kindergarten [10]. A study conducted in 3 provinces in Turkey on 7540 children reported that 62.7%, and 46.0% of the children experienced psychological, and physical abuse, respectively [11]. A study on 154 children in Afghanistan concluded that 71% of the children reported exposure to physical violence [12].

Child maltreatment affects morbidity, mortality, social behavior, wellbeing, and quality of life [11, 13, 14]. Child victimization has been shown to impair physical and mental health [15]. Researchers found a link between parental use of harsh verbal discipline and depressive symptoms, low self-esteem, and academic achievements [16, 17]. Negative discipline practices such as corporal punishment, yelling, and demonstrating disappointment studied in 6 countries have been shown to be related to internalizing problems (such as anxiety and depression) and externalizing disorders such as aggression regardless of the culture or the country [18, 19]. In addition, parental aggressiveness and negligence were shown to increase child misbehaviour and hostility [20]. Exposure to child abuse increases risk of smoking, immobility, obesity, diabetes, heart, lung and hepatic diseases [21–25]. A study conducted in Canada on child abuse in early life found a positive association between slapping and spanking children and psychiatric disorders, alcohol addiction, and dependence in later life [26].

Several factors have been shown to influence the prevalence of child abuse. Those include demographics such as age, gender, education [27, 28]. Family structure, parental conflicts, single mothers, number of children in the family, and parental warmth are important determinants of child maltreatment [29–31]. In addition, parents; economic status, family daily stressors, neighbourhood poverty, and place of residence play a major role in child abuse rates [11, 32]. A systematic review of the literature indicated that children with disabilities are more likely to be abused compared to other children with no disability [33]. In 2010, Euser and colleagues reported that children of refugee families remain at high risk of being abused or maltreated [34]. Furthermore, cultural issues, religious considerations, and ethnicity affect prevalence of violence against children [35, 36]. Indeed, research has shown that parental beliefs, attitude towards domestic violence, exposure of parents to maltreatment in their early childhood, parent’s exposure to violence, civil conflicts, presence of armed groups, armed conflicts, and political stress are major determinants of child abuse [37–40].

Political instability and war like conditions have been shown to result in the general deterioration of the quality of life of families including social, economic and developmental aspects [41, 42]. Children are more susceptible and prone to have greater negative health consequences in such environments [43]. The Palestinian context is characterized by a protracted chronic political conflict extending for several decades [44]. This conflict adds more stressors and burdens on family and parents and as has been reported elsewhere is likely to increase the chance of child abuse [37]. Children living in such an environment are very vulnerable and research on prevention and development of policy guidelines is indispensable and must be considered a top priority [45].

Research on child exposure to violence is relatively rare and inconsistent in the developing countries [46]. A study conducted in 35 low and middle income countries showed that 75% of the children aged 2 to 14 years were exposed to some forms of violent disciplines [47]. In addition, the study showed that Arabic countries reported higher rates of physical punishment, with Palestine being the most disadvantaged. Indeed, the Palestinian Central Bureau of Statistics (PCBS) conducted a Multiple Indicator Cluster Survey in collaboration with the UNICEF in 2014 and assessed some of the commonly used forms of violence (8 practices were investigated, see Index 1, V 10, 11, 15, 18, 24, 29, 31, 36) and found that 92.2% of the Palestinian children experienced one or more incidents of physical or psychological abuse in the month that preceded the survey [48]. However, no study has included internationally recognized forms, methods, and practices of child abuse and uncovered major predictors in the Palestinian context.

The theoretical framework upon which this study was designed is based on an ecological model indicating an active role of the human being in the development process and a permanent mutual influence between humans and the environment where they inhabit. This model proposes parenting practices as dependent on the child, parents and the larger socioeconomic and cultural context that eventually affect the human development process and behavior [49]. Given such a framework, the aim of this study is to assess prevalence of child abuse in the West Bank (WB) of the occupied Palestinian territory (oPt) and to determine some of its associated factors. We hypothesize that (1) the prevalence of child abuse in the WB is high and (2) child abuse has social and political determinants.

## Methods

### Data source

This study used secondary data extracted from a larger national cross sectional household survey conducted in Palestine and Qatar to compare child disciplinary methods used by caregivers in both countries using the ICAST-P tool in 2014/2015 [50]. The data is representative of the Palestinian children living in the WB and aged 0 to 12 years old. Stratified multistage cluster sampling based on the Palestinian Census Projections of 2007 was used -where the total population of the WB was 2.3 Million- taking size of the different communities of the WB in consideration. Nonresponse rate was 5.6% (with 3.6% refusal rate and 2% absence rate). One thousand five hundred ten mothers were interviewed. Questionnaires with missing data (403 cases) were not included in the final analysis. When we checked, the distribution of the missing cases was generally similar to the distribution of the sample. This was done to ensure that the missing responses were not clustered in a specific area or locality. Data analysis was performed on 1107 respondents.

### Instrument

The International Society for Prevention of Child Abuse and Neglect (ISPCAN) side by side with the UNICEF has developed ISPCAN Child Abuse Screening Tools (ICASTs). Three questionnaires were developed to estimate incidence and prevalence of global violence against children at various stages of their life [51]. In this study, a questionnaire was administered to mothers (ICAST-P) to obtain information on a range of disciplinary (both nonviolent and violent methods including physical and psychological) methods used by parents to discipline their children. This tool which includes the most commonly used disciplinary methods around the globe was used to estimate the extent of abuse of children (aged 0 to 12 years old) at home as reported by their mothers. This tool does not only uncover disciplinary practices the caregivers utilise to discipline their children, but is also a valuable tool to understand and compare the practices across several contexts, cultures, and countries [52].

### Outcome measured

The outcome investigated was child abuse assessed through exposure to physical (moderate or severe) and psychological violence. The mothers were asked about 28 most commonly used practices around the globe and which are included in the ICAST-P tool (Index 1). Internal consistency was checked and Chronbach’s alfa was 0.82 indicating very good internal consistency. A six point Likert scale (1 = once or twice, 2 = 3–5 times, 3 = 6–10 times, 4 = more than 10 times, 5 = not in the past year, 6 = never) was used to rate answer of each item. Responses were recoded as 0 (never or not in the past year) and 1(one to 10 times). The scores were summed up to form a scale then multiplied by100/28 to get a 100 point scale. The scale was recoded as low to moderate levels versus high levels of disciplinary methods. 29.66% was used as the cutoff point based on the formula mean ± 0.25 standard deviation [53]. Accordingly, those who experienced 8 or less incidents of violence during the past year were considered as with low to moderate levels of child abuse and those who experienced 9 or more incidents of violence were considered as with high levels of child abuse.

### Independent variables

The Independent variables investigated were the variables proposed by the ISPCAN questionnaire in addition to selected variables deemed necessary to cover in the Palestinian context. Study variables were child’s age and gender, Parents age, size of the family, child position in the family, parents’ levels of education, type of locality, region, refugee status (Palestinian refugees refer to those who were displaced in 1948 and 1967 and have become residents in West Bank camps since then), child disability, and dependency ratio (no. of children below 14 years old and/or elderly 65 years and above). Parental warmth measured by three questions about child being loved by his parents was also included (Index 2). Chronbach’s alfa for this scale was very good (0.84) and the factor analysis showed that the three questions of warmth are loaded on a single variable. A six point Likert scale (1 = more than 10 times, 2 = six to 10 times, 3 = three to five times, 4 = once or twice, 5 = not in the last year, 6 = never) used to rate the answers on how many times the child was told he was loved was then recoded as 0 (no, never) and 4 (more than 10 times). Answers were summed up for the three variables to form a scale from 0 (minimum warmth) to 12 (maximum warmth). The scale was then converted to 100 by multiplying the scale by 100/12. Using the same formula mean ± 0.25 standard deviation [53], the scale was categorized into low levels versus moderate to high levels of parental warmth. In addition, we have investigated exposure of the parents to political violence using the two variables: exposure to sound bombs and/or tear gas bombs during the last 12 months. A five point Likert scale (1 = yes, extremely, 2 = yes, a lot, 3 = yes, moderately, 4 = yes, a little, 5 = no, not at all) was used to rate the answers. The scale responses were recoded as 0 (no, not at all) or 1(yes little, moderate, a lot, or extreme). Answers were summed up for the two variables to form a scale with 0 (no. not at all) and 1 (yes). Chronbach’s alfa for this scale was (0.84) indicating very good internal consistency.

### Statistical analyses

The outcome was child abuse -physical and emotional- categorized into two categories low to moderate levels versus high levels. All the independent variables were used as categorical variables in the analysis. Background characteristics were investigated in the univariate analysis. Bivariate analysis to assess the association between the outcome and the independent variables was completed using Chi square test. Multivariate binary logistic regression analysis was performed with all the variables that remained significant in the bivariate analysis using SPSS version 20.

### Inclusion and exclusion criteria

Children aged 0 to 12 years and mothers aged 16 years and above were included in the study. The study was specific to children of the WB only.

## Results

Table 1 shows background characteristics of the sample population. 48.1% of the children were females, 51.3% were less than 6 years old, 21.9% of the mothers were 16–29 years old, 9.7% of the children ranked as first born child in the family, 92.9% of the families were nuclear, 60.8% of fathers had less than secondary education, 8.9% of the mothers were employed, 10.7% of the children were considered to have one or more disabilities, 30.4% of the respondents were refugees, 22.0% of the children experienced low levels of parental warmth, and 41.3% of the families were exposed to political violence.

**Table 1:** Descriptive characteristics of the study sample

Variable	Number	Percentage (%)
Gender		
Male	575	51.9
Female	532	48.1
Child’s age (years)		
Less than 6	568	51.3
6 to 9	400	36.1
10 to 12	139	12.6
Mother’s age		
16–29	242	21.9
30–39	540	48.7
40–49	284	25.7
50 and above	41	3.7
Father’s age		
16–29	61	5.5
30–39	423	38.2
40–49	457	41.3
50 and above	166	15.0
Family size		
Up to 4	177	16.0
5 to 7	660	59.6
8 and more	270	24.4
Child’s position in family.		
First/Only child	107	9.7
Middle child	325	29.4
Last child	675	60.9
Family structure		
Nuclear	1028	92.9
Extended	79	7.1
Education (mothers)		
Below secondary	648	58.5
Secondary	209	18.9.
Post-secondary	250	22.6
Education (father)		
Below secondary	673	60.8
Secondary	194	17.5
Post-secondary	240	21.7
Employment (mother)		
Employed	99	8.9
Housewife or unemployed	1008	91.1
Employment (father)		
Laborer	333	30.1
Middle class manager/landowner	656	59.3
Unemployed	118	10.7
Number of persons working part-time in household		
No body	931	84.1
1 to 4 persons	176	15.9
Dependency ratio		
No body	69	6.2
1 to 3 persons	166	15.0
4 and more	872	78.8
Child disability		
No	988	89.3
Yes	119	10.7
Locality type		
Urban	412	37.2
Rural	614	55.5
Camp	81	7.3
Region		
North WB	507	45.8
Center WB	252	22.8
South WB	348	31.4
Refugee status		
Refugee	336	30.4
Non refugee	771	69.6
Parental warmth levels		
Low	244	22.0
Moderate to high	863	78.0
Political violence		
No	650	58.7
Yes	457	41.3

Overall, 33.9% of the children were exposed to high levels of abuse as reported by their mothers (Table 2). Mean level of child abuse was 25.8% (SD ±15.4). 36.9% of the males experienced high levels of abuse compared to 30.6% of females (*p* < .05). 42.1% of the children whose mothers aged 16–29 years old were exposed to high levels of abuse compared to 12.2% of those whose mothers aged 50 years and above (*p* < .05). 37.4% of the children who were first or only children in the family were abused compared to 29.9% of those who were last in the family (*p* < .05). 33.0% of the non-disabled children experienced high levels of abuse compared to 41.2% of the disabled children (*p* < .05). 49.6% of the children with low levels of parental warmth were abused compared to 29.4% of the children with moderate to high levels of parental warmth (*p* < .05), and 30.3% of the children whose families were not exposed to political violence were abused compared to 38.9% of those who were exposed to political violence (*p* < .05).

**Table 2:** Child abuse by sociodemographic and political determinants

Variable (n)	Variable (n)	Child abuse (%) Low to moderate levels	Child abuse (%) High levels	Chi square (*P*-value)
Gender (child)	Male (575)	63.1	36.9	0.017
	Female (532)	69.4	30.6	
Children age (years)	Less than 6 (568)	65.3	34.7	0.397
	6 to 9 (400)	65.5	34.5	
	10 to 12 (139)	71.2	28.8	
Mothers age	16–29 (242)	57.9	42.1	< 0.001
	30–39 (540)	64.1	35.9	
	40–49 (284)	73.9	26.1	
	50 and above (41)	87.8	12.2	
Fathers age	19–29 (61)	60.7	39.3	0.003
	30–39 (423)	60.8	39.2	
	40–49 (457)	68.5	31.5	
	50 and above (166)	75.3	24.7	
Child’s position in Family	First/Only child (107)	62.6	37.4	0.002
	Middle child (325)	59.1	40.9	
	Last child (675)	70.1	29.9	
Education (father)	Below secondary (673)	63.9	36.1	0.007
	Secondary (194)	63.4	36.6	
	Post-secondary (240)	74.6	25.4	
Number of persons working part-time at household	No body (931)	64.8	35.2	0.017
	1 to 4 persons (176)	73.3	26.7	
Dependency ratio	No body (69)	58.0	42.0	0.019
	1–3 persons (166)	74.7	25.3	
	More than 3 (872)	65.1	34.9	
Child disability	No (988)	67.0	33.0	0.048
	Yes (119)	58.8	41.2	
Locality type	Urban (412)	66.0	34.0	0.030
	Rural (614)	67.9	32.1	
	Camp (81)	53.1	46.9	
Refugee status	Refugee (336)	61.3	38.7	0.016
	Non refugee (771)	68.2	31.8	
Parental warmth	Low (244)	50.4	49.6	< 0.001
	Moderate to high (863)	70.6	29.4	
Political violence	No (650)	69.7	30.3	0.002
	Yes (457)	61.1	38.9	
Overall		(732) 66.1	(375) 33.9	

Table 3 represent results of multivariate binary logistic regression analyses for child abuse. Males were 1.4 times as likely to be abused compared to females [OR = 1.37, 95%CI (1.05–1.78)]. Children of mothers aged 16–29 years were 5 times as likely to be abused compared to those of mothers aged 50 years and above [OR = 4.89, 95%CI (1.63–14.65)]. Children whose fathers were with low levels of education were 1.6 times as likely to be abused [OR = 1.55, 95%CI (1.01–2.38)]. Children with low levels of parental warmth were twice as likely to be abused [OR = 2.06, 95%CI (1.50–2.83)], and children whose parents were exposed to political violence were 1.4 times as likely to be abused [OR = 1.39, 95%CI (1.06–1.82)].

**Table 3:** Multivariate binary logistic regression analysis for levels of child abuse

Variable		***P*** value	Adjusted OR	95% C.I.	
				Lower	Upper
Gender	Female		1		
	Male	0.021	1.37	1.05	1.78
Mother’s age	50 and above	0.017	1		
	16–29	0.005	4.89	1.63	14.65
	30–39	0.015	3.64	1.28	10.31
	40–49	0.069	2.56	0.93	7.06
Father’s education	Post-secondary	0.098	1		
	Less than secondary	0.064	1.40	0.98	1.98
	Secondary	0.045	1.55	1.01	2.38
Parental warmth	Moderate to high		1		
	Low	<0.001	2.06	1.50	2.83
Exposure to political violence	No		1		
	Yes	0.017	1.39	1.06	1.82

## Discussion

Child abuse is prevalent among children in the WB of the Palestinian society. This high prevalence was reported in a previous study which indicated that 92.2% of the Palestinian children experienced one or more incidents of violence by their mothers during the month that preceded the survey [48]. Overall, 33.9% of the children in this study experienced high levels (9 or more incidents) of physical and psychological/emotional abuse during the past year as reported by their mothers. This finding is also comparable with other reported studies from Jordan, Syria, Iraq, and Turkey [11, 27, 54].

Our study found that males were more likely to be abused compared to females. This finding is supported by other studies which reported that male children were more likely to be physically [55] and psychologically abused [56, 57]. For instance, our data showed that 61.7% of the children were spanked (Index 1, V24) with males being more disadvantaged than females (65.6% for males versus 57.5% for females). This trend could be attributed to the patriarchal system prevalent in our society as has been reported elsewhere [27]. In 1991, Lytton and Romney in their meta-analysis reported that male children are usually more resistant and express more disobedient bahaviors compared to female children and hence more likely to be physically punished [58]. In addition, some researchers attributed this trend to biological factors and to the gender roles that children are socialized into where boys are given more freedom, autonomy, and power than girls who are treated more strictly and taught to be more obedient than boys early on [59, 60]. Akmatov investigated child abuse in 28 developing countries and reported that the higher prevalence of child abuse among boys could be attributed to higher expectations from boys since they are seen as the future family’s main source of income [61]. No statistically significant differences were detected by child’s age in this study.

Younger mothers were more likely to abuse their children compared to older mothers. This finding endorses findings of other studies which provided evidence on this trend [62, 63]. Researchers attributed this to life experience such as lack of knowledge and experience in child rearing among younger mothers [64]. Indeed, Whitman and colleagues (as reported by Bert and colleagues) concluded that younger and adolescent mothers were more likely to be intolerant, insensitive, and impatient making them more eligible to involve negative discipline practices than older mothers [65]. In addition, as age advances, mothers develop more parenting skills which is likely to have positive impact on their behavior and attitudes [66].

Education of the father was an important predictor of child abuse. High levels of father’s education were associated with less use of harsh disciplinary methods while no effect of mother education was observed. Similar findings were reported in Turkey in the recent years [27]. Indeed, levels of education are important predictors of socioeconomic status of the family [67]. Research has shown that lower family socioeconomic situation, resulting in poverty and economic hardships, is an important precursor of child maltreatment [68, 69]. On the other hand higher education level with its role in improving socioeconomic status enables people to fulfil their daily needs and respond to demands, relieving stress, and stabilizing the family. In addition education protects parents from involvement in negative bahaviors such as cigarette smoking and alcohol consumption [69–71]. This could be an explanation of why highly educated fathers are less likely to commit child abuse.

Our result showed that Low levels of parental warmth increase risk of child abuse. This finding endorses findings of Wade and Kendler who reported an inverse association between parental warmth and child abuse [72]. This finding is attributed to the fact that parents who show high levels of parental warmth are more likely to utilize peaceful communication and interaction with their children [73] which is well reported to be protective against child abuse [74, 75]. Better social and emotional child development were reported among parents who showed love, support, and acceptance to their children. In contrast, inferior social and emotional child development were reported for children whose parents exercised punishment and rejection [76].

Exposure of parents to political violence was associated with increased chances of child abuse. Research has shown that exposure to contextual violence and existence of armed groups in communities were associated with increased violent disciplines such as corporal punishment [37]. In fact, exposure to political violence and conflicts have negative consequences on people’s life such as suffering, distress, trauma, general and mental health, family stability and functioning, and wellbeing of the communities in general [77, 78]. The resulting deterioration of social, economic, and political structures has been well reported as increasing the risk of child maltreatment [71, 79]. In addition, Cuartas and colleagues in 2019 reported that exposure to violence and armed conflicts is likely to alter dynamics, social norms, and attitudes towards the use of violent discipline [37].

### Study limitations

Despite being conducted on a regionally representative sample, our study has got some limitations. This is a cross sectional study that elucidates association but not causation. Important variables like parents’ wellbeing, distress, and conflict were not investigated. Mothers may have underreported some of the violent practices to avoid pressure from the family and the community. This study did not include Palestinian children of the Gaza Strip.

## Study implications

Children have the right of protection from all forms of violence as has been stipulated by the Convention on the Rights of the Child (CRC) [47]. The findings of this study are very important and provide guidance to policy makers to identify high risk groups among children where preventive interventions are essential and constitute a top priority. Such interventions are necessary for creating an optimum environment for a healthy and safe development of children in the early stage of life so that they can achieve their maximum potential. These interventions should be implemented side by side with the principles of child rights illustrated in the CRC without compromising the capacity of families to exercise the desired harmless forms of child discipline. Providing young mothers with education and support in the use of appropriate rearing practices together with an understanding of the consequences of child abuse could be important steps in preventing violence against children. Those providing the education and support, such as health and social care providers will themselves require training and on-going support. Palestine has ratified the CRC recently and policy makers need to make concerted efforts to protect our future generations. Thus, laws and legislations should be set to ensure an optimum environment for the development and protection of the child. A just political solution to the Palestine question that ends the protracted conflict and exposure to violence is essential if we are to save our children and improve their lives and wellbeing.

## Conclusions

Our results revealed an overall high prevalence of child abuse in the WB of the oPt. The most disadvantaged children were boys, children whose mothers were younger, children whose fathers were less educated, children with low levels of parental nurturing, and children whose parents were exposed to political violence. These findings identify and emphasize the place of social, economic, and political issues as major determinants of family wellbeing and population health.

## Supplementary information

**Additional file 1: Index 1.** Physical and emotional child abuse questions (adapted from ICAST-P questionnaire).

**Additional file 2: Index 2.** Parental nurturing questions (developed by Samia Halileh, a senior Palestinian paediatrician, from her local research and practice).

## Data Availability

This study used secondary data extracted from a larger national cross sectional household survey conducted in Palestine and Qatar to compare child disciplinary methods used by caregivers in both countries using the ICAST-P tool in 2014/2015. Two of the authors of this paper (Rita Giacaman and Margaret Lynch) were involved in the original study design. Thus, they have a legal access to the data and no permission or consent was required. The data is not available publicly. However, upon a reasonable request, the data can be obtained from the authors.

## Abbreviations

UNICEF: United Nations International Children’s Emergency Fund
PCBS: Palestinian Central Bureau of Statistics
WB: West Bank
oPt: Occupied Palestinian territory
ISPCAN: The International Society for Prevention of Child Abuse and Neglect
ICASTs: ISPCAN Child Abuse Screening Tools
ICAST-P: ISPCAN Child Abuse Screening Tool (parents version)
95%CI: 95% confidence intervals
OR: Odds ratios
CRC: Convention on the Rights of the Child
