# Supplementary material for: Child abuse in the West Bank of the occupied Palestinian territory (WB/oPt): social and political determinants
Source: BMC Public Health. 2020 Jul 18;20:1130. doi: 10.1186/s12889-020-09251-x (PMC7368693; doi:10.1186/s12889-020-09251-x)
Supplement: Supplementary file 1 — Additional file 1: Index 1. Physical and emotional child abuse questions (adapted from ICAST-P questionnaire). [file 12889_2020_9251_MOESM1_ESM.docx]

**Index 1: Physical and emotional child abuse questions** (adapted from ICAST-P questionnaire)

V10. “Shook him/her”

V11. “Hit him or her on the buttocks with an object such as a shoe, stick, broom, cane or belt”

V12. “Hit elsewhere (not buttocks) with an object such as a shoe, stick, broom, cane or belt”

V14. “Twisted his/her ear”

V15. “Hit him/her on head with knuckle or back of the hand”

V16. “Pulled his/her hair”

V17. “Threatened to leave or abandon him/her”

V18. “Shouted, yelled, or screamed at him/her”

V19. “Threatened to invoke ghosts, ghoul or evil spirits, or harmful people”

V20. “Kicked him/her with a foot”

V21. “Put chili pepper, hot pepper, or spicy food in mouth (to cause pain)”

V22. “Forced him/her to kneel or stand in a manner that result in pain”

V23. “Cursed him/her (Son of a…)”

V24. “Spanked him/her on the bottom with bare hand”

V25. “Choked him/her or squeezed his or her neck with hands (or something else)”

V26. “Threatened to kick out of house or send away for a long time”

V27. “Locked out of the house”

V29. “Insulted him/her by calling [name] dumb, lazy, or other names like that”

V30. “Pinched him/her”

V31. “Slapped on face or back of head”

V32. “Refused to speak to him/her”

V33. “Withheld a food meal as punishment”

V34. “Used a hand or pillow to cover the mouth and nose to prevent breathing (smother)”

V35. “Burned, scalded or branded him/her”

V36. “Hit him or her over and over again with object or fist (“beat-up”)”

V37. “Threatened him/her with a knife or gun”

V38. “Locked him or her in a dark room”

V39. “Used public humiliation to discipline him or her”
