# Supplementary material for: Child abuse in the West Bank of the occupied Palestinian territory (WB/oPt): social and political determinants
Source: BMC Public Health. 2020 Jul 18;20:1130. doi: 10.1186/s12889-020-09251-x (PMC7368693; doi:10.1186/s12889-020-09251-x)
Supplement: Supplementary file 2 — Additional file 2: Index 2. Parental nurturing questions (developed by Samia Halileh, a senior Palestinian paediatrician, from her local research and practice). [file 12889_2020_9251_MOESM2_ESM.docx]

**Index 2: Parental nurturing questions** (developed by Samia Halileh, a senior Palestinian paediatrician, from her local research and practice)

P21- “Did you/husband/care taker tell this child that you loved him during the past 12 months?”

P22- “Did you ever show love to this child during the past 12 months?”

P24- “Do you think this child knows that he/she is loved?”
